# International Society of Sports Nutrition position stand: safety and efficacy of creatine supplementation in exercise, sport, and medicine

**DOI:** 10.1186/s12970-017-0173-z

**Published:** 2017-06-13

**Authors:** Richard B. Kreider, Douglas S. Kalman, Jose Antonio, Tim N. Ziegenfuss, Robert Wildman, Rick Collins, Darren G. Candow, Susan M. Kleiner, Anthony L. Almada, Hector L. Lopez

**Affiliations:** 1 Exercise & Sport Nutrition Lab, Human Clinical Research Facility, Department of Health & Kinesiology, Texas A&M University, College Station, TX 77843-4243 USA; 2 Nutrition Research Unit, QPS, 6141 Sunset Drive Suite 301, Miami, FL 33143 USA; 3 Department of Health and Human Performance, Nova Southeastern University, Davie, FL 33328 USA; 4 The Center for Applied Health Sciences, 4302 Allen Road, STE 120, Stow, OH 44224 USA; 5 Post Active Nutrition, 111 Leslie St, Dallas, TX 75208 USA; 6 Collins Gann McCloskey & Barry, PLLC, 138 Mineola Blvd., Mineola, NY 11501 USA; 7 Faculty of Kinesiology and Health Studies, University of Regina, Regina, SK S4S 0A2 Canada; 8 High Performance Nutrition, LLC, Mercer Island, WA 98040 USA; 9 Vitargo Global Sciences, Inc., Dana Point, CA 92629 USA; 10 Supplement Safety Solutions, LLC, Bedford, MA 01730 USA

**Keywords:** Ergogenic aids, Performance enhancement, Sport nutrition, Athletes, Muscular strength, Muscle power, Clinical applications, Safety, Children, Adolescents

## Abstract

Creatine is one of the most popular nutritional ergogenic aids for athletes. Studies have consistently shown that creatine supplementation increases intramuscular creatine concentrations which may help explain the observed improvements in high intensity exercise performance leading to greater training adaptations. In addition to athletic and exercise improvement, research has shown that creatine supplementation may enhance post-exercise recovery, injury prevention, thermoregulation, rehabilitation, and concussion and/or spinal cord neuroprotection. Additionally, a number of clinical applications of creatine supplementation have been studied involving neurodegenerative diseases (e.g., muscular dystrophy, Parkinson’s, Huntington’s disease), diabetes, osteoarthritis, fibromyalgia, aging, brain and heart ischemia, adolescent depression, and pregnancy. These studies provide a large body of evidence that creatine can not only improve exercise performance, but can play a role in preventing and/or reducing the severity of injury, enhancing rehabilitation from injuries, and helping athletes tolerate heavy training loads. Additionally, researchers have identified a number of potentially beneficial clinical uses of creatine supplementation. These studies show that short and long-term supplementation (up to 30 g/day for 5 years) is safe and well-tolerated in healthy individuals and in a number of patient populations ranging from infants to the elderly. Moreover, significant health benefits may be provided by ensuring habitual low dietary creatine ingestion (e.g., 3 g/day) throughout the lifespan. The purpose of this review is to provide an update to the current literature regarding the role and safety of creatine supplementation in exercise, sport, and medicine and to update the position stand of International Society of Sports Nutrition (ISSN).

## Background

Creatine is one of the most popular nutritional ergogenic aids for athletes. Studies have consistently shown that creatine supplementation increases intramuscular creatine concentrations, can improve exercise performance, and/or improve training adaptations. Research has indicated that creatine supplementation may enhance post-exercise recovery, injury prevention, thermoregulation, rehabilitation, and concussion and/or spinal cord neuroprotection. A number of clinical applications of creatine supplementation have also been studied involving neurodegenerative diseases (e.g., muscular dystrophy, Parkinson’s, Huntington’s disease), diabetes, osteoarthritis, fibromyalgia, aging, brain and heart ischemia, adolescent depression, and pregnancy. The purpose of this review is to provide an update to the current literature regarding the role and safety of creatine supplementation in exercise, sport, and medicine and to update the position stand of International Society of Sports Nutrition (ISSN) related to creatine supplementation.

## Metabolic role

**Fig. 1:**
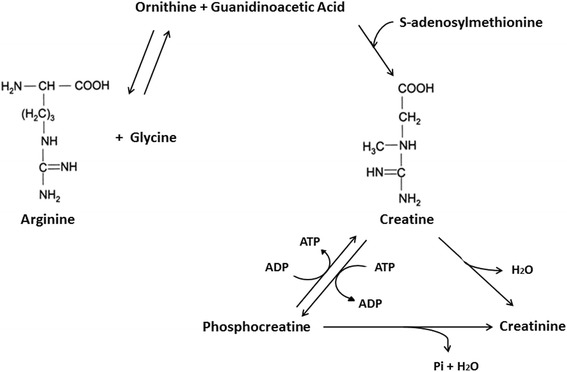
Chemical structure and biochemical pathway for creatine synthesis. From Kreider and Jung [6]

Creatine, a member of the guanidine phosphagen family, is a naturally occurring non-protein amino acid compound found primarily in red meat and seafood [1–4]. The majority of creatine is found in skeletal muscle (~95%) with small amounts also found in the brain and testes (~5%) [5, 6]. About two thirds of intramuscular creatine is phosphocreatine (PCr) with the remaining being free creatine. The total creatine pool (PCr + Cr) in the muscle averages about 120 mmol/kg of dry muscle mass for a 70 kg individual [7]. However, the upper limit of creatine storage appears to be about 160 mmol/kg of dry muscle mass in most individuals [7, 8]. About 1–2% of intramuscular creatine is degraded into creatinine (metabolic byproduct) and excreted in the urine [7, 9, 10]. Therefore, the body needs to replenish about 1–3 g of creatine per day to maintain normal (unsupplemented) creatine stores depending on muscle mass. About half of the daily need for creatine is obtained from the diet [11]. For example, a pound of uncooked beef and salmon provides about 1–2 g of creatine [9]. The remaining amount of creatine is synthesized primarily in the liver and kidneys from arginine and glycine by the enzyme arginine:glycine amidinotransferase (AGAT) to guanidinoacetate (GAA), which is then methylated by guanidinoacetate N-methyltransferase (GAMT) using S-adenosyl methionine to form creatine (see Fig. 1) [12].

Some individuals have been found to have creatine synthesis deficiencies due to inborn errors in AGAT, GMAT and/or creatine transporter (CRTR) deficiencies and therefore must depend on dietary creatine intake in order to maintain normal muscle and brain concentrations of PCr and Cr [13–19]. Vegetarians have been reported to have lower intramuscular creatine stores (90–110 mmol/kg of dry muscle) and therefore may observe greater gains in muscle creatine content from creatine supplementation [11, 13, 20, 21]. Conversely, larger athletes engaged in intense training may need to consume 5–10 g/day of creatine to maintain optimal or capacity whole body creatine stores [22] and clinical populations may need to consume 10–30 g/day throughout their lifespan to offset creatine synthesis deficiencies and/or provide therapeutic benefit in various disease states [13, 19, 23].

**Fig. 2:**
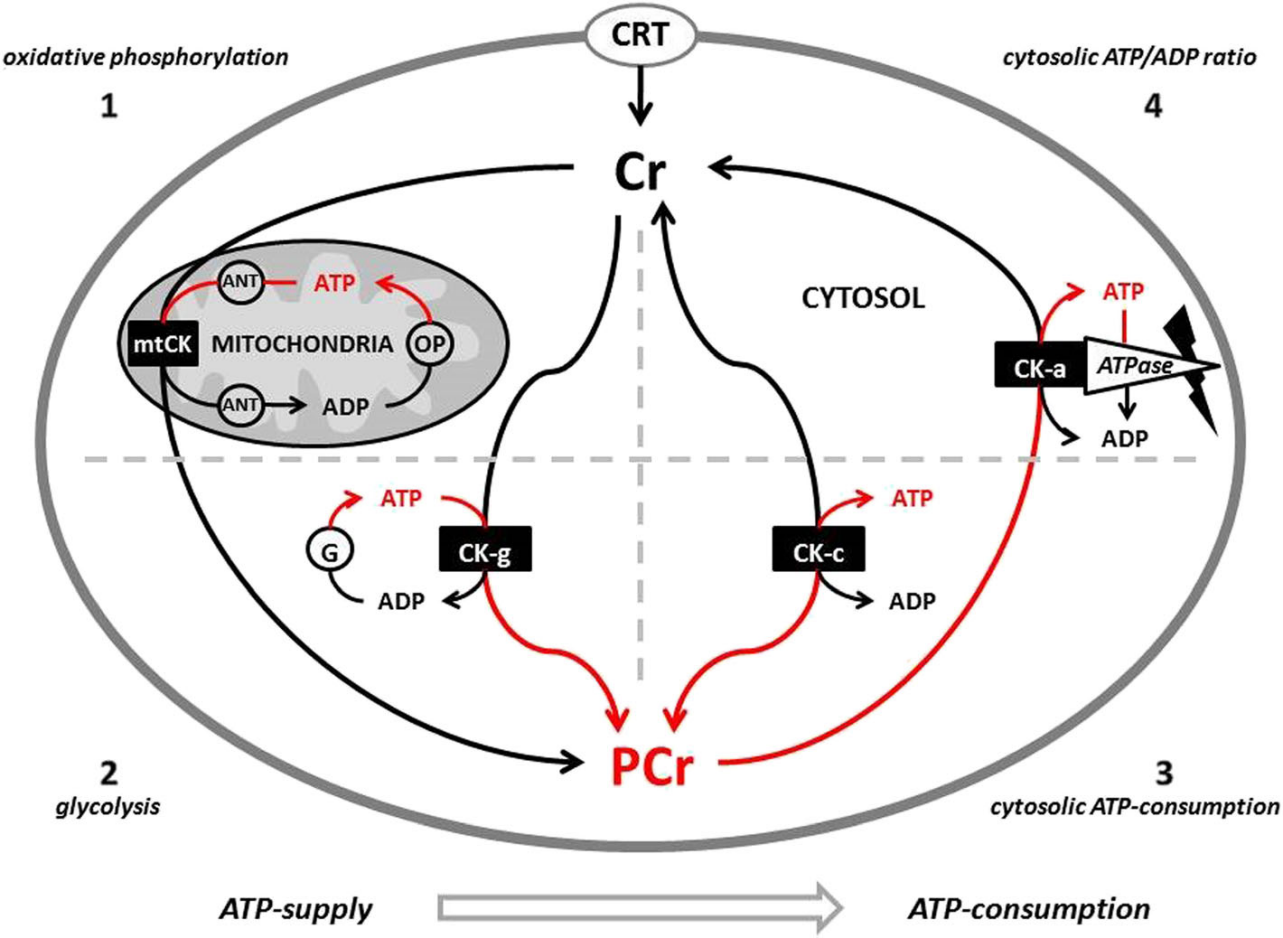
Proposed creatine kinase/phosphocreatine (CK/PCr) energy shuttle. CRT = creatine transporter; ANT = adenine nucleotide translocator; ATP = adenine triphosphate; ADP = adenine diphosphate; OP = oxidative phosphorylation; mtCK = mitochondrial creatine kinase; G = glycolysis; CK-g = creatine kinase associated with glycolytic enzymes; CK-c = cytosolic creatine kinase; CK-a = creatine kinase associated with subcellular sites of ATP utilization; 1 – 4 sites of CK/ATP interaction. From Kreider and Jung [6]

**Fig. 3:**
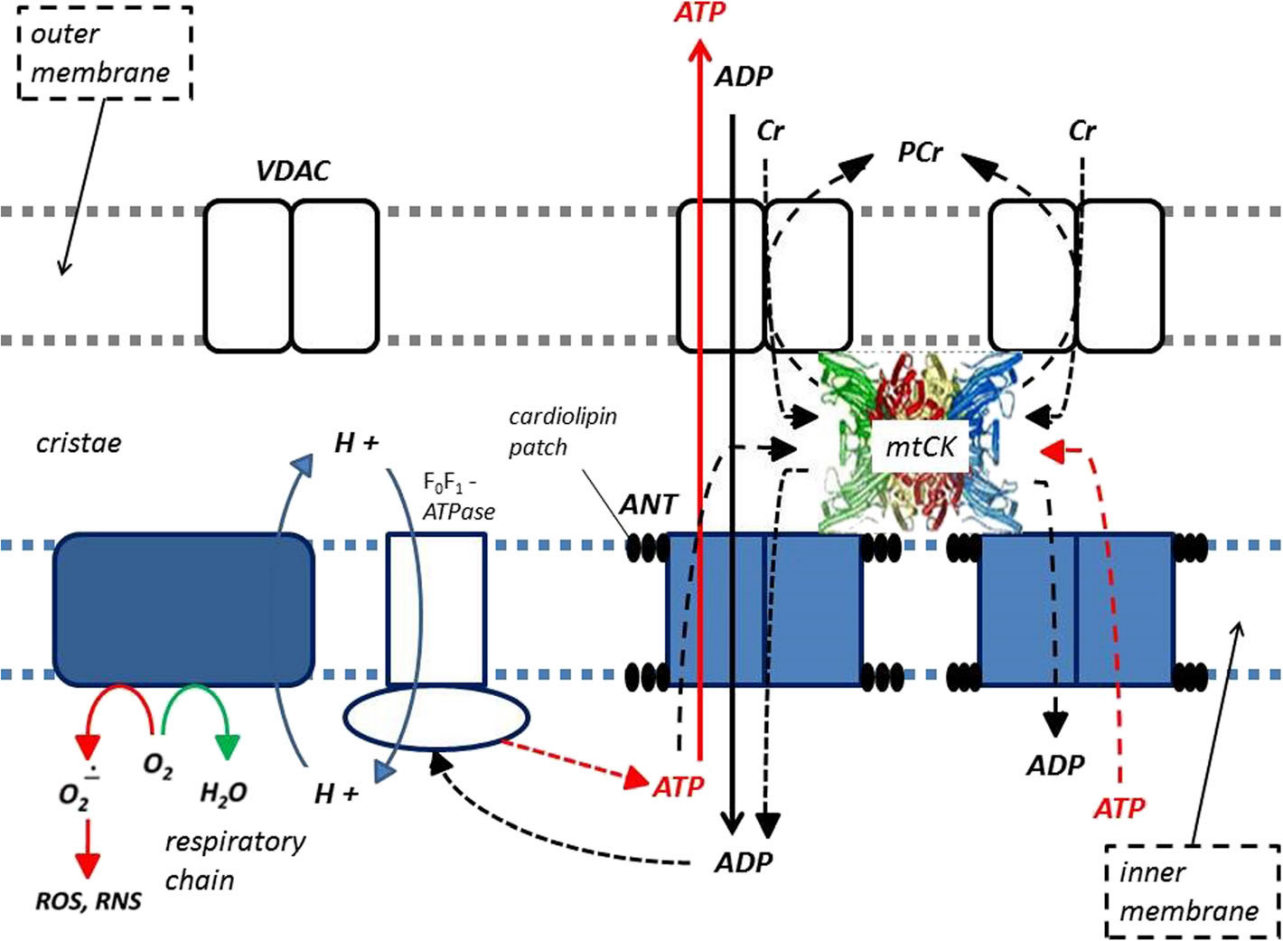
Role of mitochondrial creatine kinase (mtCK) in high energy metabolite transport and cellular respiration. VDAC = voltage-dependent anion channel; ROS = reactive oxygen species; RNS = reactive nitrogen species; ANT = adenine nucleotide translocator; ATP = adenine triphosphate; ADP = adenine diphosphate; Cr = creatine; and, PCr = phosphocreatine. From Kreider and Jung [6]

Phosphagens are prevalent in all species and play an important role in maintaining energy availability [1, 2, 24, 25]. The primary metabolic role of creatine is to combine with a phosphoryl group (Pi) to form PCr through the enzymatic reaction of creatine kinase (CK). Wallimann and colleagues [26–28] suggested that the pleiotropic effects of Cr are mostly related to the functions of CK and PCr (i.e., CK/PCr system). As adenosine triphosphate (ATP) is degraded into adenosine diphosphate (ADP) and Pi to provide free energy for metabolic activity, the free energy released from the hydrolysis of PCr into Cr + Pi can be used as a buffer to resynthesize ATP [24, 25]. This helps maintain ATP availability particularly during maximal effort anaerobic sprint-type exercise. The CK/PCr system also plays an important role in shuttling intracellular energy from the mitochondria into the cytosol (see Fig. 2). The CK/PCr energy shuttle connects sites of ATP production (glycolysis and mitochondrial oxidative phosphorylation) with subcellular sites of ATP utilization (ATPases) [24, 25, 27]. In this regard, creatine enters the cytosol through a CRTR [16, 29–31]. In the cytosol, creatine and associated cytosolic and glycolytic CK isoforms help maintain glycolytic ATP levels, the cytosolic ATP/ADP ratio, and cytosolic ATP-consumption [27]. Additionally, creatine diffuses into the mitochondria and couples with ATP produced from oxidative phosphorylation and the adenine nucleotide translocator (ANT) via mitochondrial CK (see Fig. 3). ATP and PCr can then diffuse back into the cytosol and help buffer energy needs. This coupling also reduces the formation of reactive oxygen species (ROS) and can therefore act as a direct and/or indirect antioxidant [32–35]. The CK/PCr energy shuttle thereby connects sites of ATP production (glycolysis and mitochondrial oxidative phosphorylation) with subcellular sites of ATP utilization (ATPases) in order to fuel energy metabolism [24, 25, 27]. In this way, the CK/PCr system thereby serves as an important regulator of metabolism which may help explain the ergogenic and potential therapeutic health benefits of creatine supplementation [4, 27, 33, 36–45].

## Supplementation protocols

**Fig. 4:**
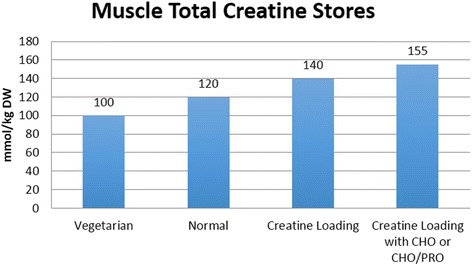
Approximate muscle total creatine levels in mmol/kg dry weight muscle reported in the literature for vegetarians, individuals following a normal diet, and in response to creatine loading with or without carbohydrate (CHO) or CHO and protein (PRO). From Kreider and Jung [6]

In a normal diet that contains 1–2 g/day of creatine, muscle creatine stores are about 60–80% saturated. Therefore, dietary supplementation of creatine serves to increase muscle creatine and PCr by 20–40% (see Fig. 4.) [7, 8, 10, 46–48]. The most effective way to increase muscle creatine stores is to ingest 5 g of creatine monohydrate (or approximately 0.3 g/kg body weight) four times daily for 5–7 days [7, 10]. However, higher levels of creatine supplementation for longer periods of time may be needed to increase brain concentrations of creatine, offset creatine synthesis deficiencies, or influence disease states [13, 19, 23]. Once muscle creatine stores are fully saturated, creatine stores can generally be maintained by ingesting 3–5 g/day, although some studies indicate that larger athletes may need to ingest as much as 5–10 g/day in order to maintain creatine stores [7, 8, 10, 46–48]. Ingesting creatine with carbohydrate or carbohydrate and protein have been reported to more consistently promote greater creatine retention [8, 22, 49, 50]. An alternative supplementation protocol is to ingest 3 g/day of creatine monohydrate for 28 days [7]. However, this method would only result in a gradual increase in muscle creatine content compared to the more rapid loading method and may therefore have less effect on exercise performance and/or training adaptations until creatine stores are fully saturated. Research has shown that once creatine stores in the muscle are elevated, it generally takes 4–6 weeks for creatine stores to return to baseline [7, 48, 51]. Additionally, it has been recommended that due to the health benefits of creatine, individuals should consume about 3 g/day of creatine in their diet particularly as one ages [27]. No evidence has suggested that muscle creatine levels fall below baseline after cessation of creatine supplementation; therefore, the potential for long-term suppression of endogenous creatine synthesis does not appear to occur [22, 52].

## Bioavailability

The most commonly studied form of creatine in the literature is creatine monohydrate [53]. The uptake of creatine involves the absorption of creatine into the blood and then uptake by the target tissue [53]. Plasma levels of creatine typically peak at about 60 min after oral ingestion of creatine monohydrate [7]. An initial rise in plasma creatine levels, followed by a reduction in plasma levels can be used to indirectly suggest increased uptake into the target tissue [53]. However, the gold standards for measuring the effects of creatine supplementation on target tissues are through magnetic resonance spectroscopy (MRS), muscle biopsy, stable isotope tracer studies, and/or whole body creatine retention assessed by measuring the difference between creatine intake and urinary excretion of creatine [53].

Creatine is stable in solid form but not in aqueous solution due to an intramolecular cyclization [54]. Generally, creatine is converted to creatinine at higher rates the lower the pH and the higher the temperature. For example, research has shown that creatine is relatively stable in solution at neutral pH (7.5 or 6.5). However, after 3 days of storage at 25 °C, creatine degrades to creatinine (e.g., 4% at pH 5.5; 12% at pH 4.5; and 21% at pH 3.5) [53, 55]. The degradation of creatine into creatinine over time is the main reason that creatine is sold in solid form. However, this does not mean that creatine is degraded into creatinine in vivo through the digestive process. In this regard, the degradation of creatine to creatinine can be reduced or halted be either lowering the pH under 2.5 or increasing the pH [53]. A very low pH results in the protonation of the amide function of the creatine molecule, thereby preventing the intra-molecular cyclization [53]. Therefore, the conversion of creatine to creatinine in the gastrointestinal tract is minimal regardless of transit time; absorption into the blood is nearly 100% [10, 53, 56, 57].

The vast majority of studies assessing the efficacy of creatine supplementation on muscle phosphagen levels, whole body creatine retention, and/or performance have evaluated creatine monohydrate. Claims that different forms of creatine are degraded to a lesser degree than creatine monohydrate in vivo or result in a greater uptake to muscle are currently unfounded [53]. Clinical evidence has not demonstrated that different forms of creatine such as creatine citrate [50], creatine serum [58], creatine ethyl ester [59], buffered forms of creatine [60], or creatine nitrate [61] promote greater creatine retention than creatine monohydrate [53].

## Ergogenic value

Table 1 presents the reported ergogenic benefits of creatine supplementation. A large body of evidence now indicates that creatine supplementation increases muscle availability of creatine and PCr and can therefore enhance acute exercise capacity and training adaptations in adolescents [62–66], younger adults [61, 67–77] and older individuals [5, 40, 43, 78–85]. These adaptations would allow an athlete to do more work over a series of sets or sprints leading to greater gains in strength, muscle mass, and/or performance due to an improvement in the quality of training. Table 2 presents the types of sport events in which creatine supplementation has been reported to benefit. Creatine supplementation has primarily been recommended as an ergogenic aid for power/strength athletes to help them optimize training adaptations or athletes who need to sprint intermittently and recover during competition (e.g., American football, soccer, basketball, tennis, etc.). After creatine loading, performance of high intensity and/or repetitive exercise is generally increased by 10–20% depending on the magnitude of increase in muscle PCr [46].

**Table 1:** Potential ergogenic benefits of creatine supplementation

• Increased single and repetitive sprint performance • Increased work performed during sets of maximal effort muscle contractions • Increased muscle mass & strength adaptations during training • Enhanced glycogen synthesis • Increased anaerobic threshold • Possible enhancement of aerobic capacity via greater shuttling of ATP from mitochondria • Increased work capacity • Enhanced recovery • Greater training tolerance	

Adapted from Kreider and Jung [6]

**Table 2:** Examples of sport events that may be enhanced by creatine supplementation

*Increased PCr* • Track sprints: 60–200 m • Swim sprints: 50 m • Pursuit cycling *Increased PCr Resynthesis* • Basketball • Field hockey • America Football • Ice hockey • Lacrosse • Volleyball *Reduced Muscle Acidosis* • Downhill skiing • Water Sports (e.g., Rowing, Canoe, Kayak, Stand-Up Paddling) • Swim events: 100, 200 m • Track events: 400, 800 m • Combat Sports (e.g., MMA, Wrestling, Boxing, etc.) *Oxidative Metabolism* • Basketball • Soccer • Team handball • Tennis • Volleyball • Interval Training in Endurance Athletes *Increased Body Mass/Muscle Mass* • American Football • Bodybuilding • Combat Sports (e.g., MMA, Wrestling, Boxing, etc.) • Powerlifting • Rugby • Track/Field events (Shot put; javelin; discus; hammer throw) • Olympic Weightlifting	

Adapted from Williams, Kreider, and Branch [269]

Benefits have been reported in men and women, although the majority of studies have been conducted on men and some studies suggest that women may not see as much gain in strength and/or muscle mass during training in response to creatine supplementation [20, 51, 64, 86–90]. However, as will be described below, a number of other applications in sport may benefit athletes involved in high intensity intermittent and endurance events as well. In terms of performance, the International Society of Sports Nutrition (ISSN) has previously concluded in its position stand on creatine supplementation that creatine monohydrate is the most effective ergogenic nutritional supplement currently available to athletes in terms of increasing high-intensity exercise capacity and lean body mass during training [5, 78]. Recent position stands by the American Dietetic Association, Dietitians of Canada, and the American College of Sports Medicine on nutrition for athletic performance all drew similar conclusions [91, 92]. Thus, a wide-spread consensus now exists in the scientific community that creatine supplementation can serve as an effective nutritional ergogenic aid that may benefit athletes involved in numerous sports as well as individuals involved in exercise training.

## Prevalence of use in sport

Creatine is found in high amounts in the food supply and therefore its use is not banned by any sport organization although some organizations prohibit provision of some types of dietary supplements to athletes by their teams [5, 53, 78, 91, 92]. In these instances, athletes can purchase and use creatine on their own without penalty or violation of their banned substance restrictions. Americans consume over four million kilograms (kg) a year of creatine with worldwide use much higher [53]. The reported prevalence of creatine use among athletes and military personnel in survey-based studies has generally been reported to be about 15–40% [93–101], with use more common in male strength/power athletes. High school athletes have been reported to have similar prevalence of use of creatine [95–97, 102]. In 2014, the NCAA reported that creatine was among the most popular dietary supplements taken by their male athletes (e.g., baseball - 28.1%, basketball - 14.6%, football - 27.5%, golf - 13.0%, ice hockey - 29.4%, lacrosse - 25.3%, soccer 11.1%, swimming - 19.2%, tennis - 12.9%, track and field - 16.1%, wrestling - 28.5%) while female athletes reported a use rate of only 0.2 to 3.8% in various sports [103]. Comparatively, these NCAA athletes reported relatively high alcohol (83%), tobacco (10–16%), and marijuana (22%) use along with minimal androgenic anabolic steroid use (0.4%). As will be noted below, no study has reported any adverse or ergolytic effect of short- or long-term creatine supplementation while numerous studies have reported performance and/or health benefits in athletes and individuals with various diseases. Therefore, the prevalence of alcohol, tobacco and drug use among NCAA athletes would seemingly be a much greater health concern than athletes taking creatine.

## Other applications in sport and training

Recent research demonstrates a number of other applications of creatine supplementation that may benefit athletes involved in intense training and individuals who want to enhance training adaptations. For example, use of creatine during training may enhance recovery, reduce the risk of injury and/or help individuals recover from injuries at a faster rate. The following describes some applications of creatine in addition to serving as an ergogenic aid.

### Enhanced recovery

Creatine supplementation can help athletes recover from intense training. For example, Green and coworkers [8] reported that co-ingesting creatine (5 g) with large amounts of glucose (95 g) enhanced creatine *and* carbohydrate storage in muscle. Additionally, Steenge et al. [49] reported that co-ingesting creatine (5 g) with 47–97 g of carbohydrate and 50 g of protein enhanced creatine retention. Nelson and colleagues [104] reported that creatine loading prior to performing an exhaustive exercise bout and glycogen loading promoted greater glycogen restoration than just carbohydrate loading alone. Since glycogen replenishment is important to promoting recovery and preventing overtraining during intensified training periods [78], creatine supplementation may help athletes who deplete large amounts of glycogen during training and/or performance to maintain optimal glycogen levels.

Evidence also suggests that creatine supplementation may reduce muscle damage and/or enhance recovery from intense exercise. For example, Cooke and associates [105] evaluated the effects of creatine supplementation on muscle force recovery and muscle damage following intense exercise. They reported that participants supplemented with creatine had significantly greater isokinetic (+10%) and isometric (+21%) knee extension strength during recovery from exercise-induced muscle damage. Additionally, plasma CK levels were significantly lower (−84%) after 2, 3, 4, and 7 days of recovery in the creatine supplemented group compared to controls. The authors concluded that creatine improved the rate of recovery of knee extensor muscle function after injury. Santos and coworkers [106] evaluated the effects of creatine loading in experienced marathon runners prior to performing a 30 km race on inflammatory markers and muscle soreness. The researchers reported that creatine loading attenuated the changes in CK (−19%), prostaglandin E2 (−61%), and tumor necrosis factor (TNF) alpha (−34%) and abolished the increase in lactate dehydrogenase (LDH) compared to controls. Similar findings were reported by Demince et al. [107] who reported that creatine supplementation inhibited the increase of inflammatory markers (TNF-alpha and C-reactive protein) in response to intermittent anaerobic sprint exercise. Finally, Volek and colleagues [77] evaluated the effects of creatine supplementation (0.3 g/kg/d) for 4 weeks during an intensified overreaching period followed by a 2 weeks taper. The researchers found that creatine supplementation was effective in maintaining muscular performance during the initial phase of high-volume resistance training overreaching that otherwise results in small performance decrements. These findings suggest that creatine supplementation can help athletes tolerate heavy increases in training volume. Therefore, there is strong evidence that creatine supplementation can help athletes enhance glycogen loading; experience less inflammation and/or muscle enzyme efflux following intense exercise; and tolerate high volumes of training and/or overreaching to a greater degree thereby promoting recovery.

### Injury prevention

Several studies have reported that creatine supplementation during training and/or competition either has no effect or reduces the incidence of musculoskeletal injury, dehydration, and/or muscle cramping. For example, several initial studies on creatine supplementation provided 15–25 g/day of creatine monohydrate for 4 – 12 weeks in athletes engaged in heavy training with no reported side effects [67, 77, 108–110]. Kreider and colleagues [109] reported that American collegiate football players ingesting 20 or 25 g/day of creatine monohydrate with a carbohydrate/protein supplement for 12 weeks during off season conditioning and spring football practice experienced greater gains in strength and muscle mass with no evidence of any adverse side effects. Additionally, in a study specifically designed to assess the safety of creatine supplementation, American collegiate football players ingesting about 16 g/day of creatine for 5 days and 5–10 g/day for 21 months had no clinically significant differences among creatine users and controls in markers of renal function, muscle and liver enzymes, markers of catabolism, electrolytes, blood lipids, red cell status, lymphocytes, urine volume, clinical urinalysis, or urine specific gravity [22]. Meanwhile, creatine users experienced less incidence of cramping, heat illness/dehydration, muscle tightness, muscle strains/pulls, non-contact injuries, and total injuries/missed practices than those not taking creatine [111].

Similar findings were reported by Greenwood and coworkers [112] who examined injury rates during a 4 months American collegiate football season among creatine users (0.3 g/kg/day for 5 days, 0.03 g/kg/day for 4 months) and non-users. The researchers reported that creatine users experienced significantly less incidence of muscle cramping, heat illness/dehydration, muscle tightness, muscle strains, and total injuries compared to athletes who did not supplement their diet with creatine. Likewise, Cancela and associates [113] reported that creatine supplementation (15 g/day x 7-d, 3 g/day x 49-d) during soccer training promoted weight gain but that those taking creating had no negative effects on blood and urinary clinical health markers. Finally, Schroder et al. [114] evaluated the effects of ingesting creatine (5 g/day) for three competitive seasons in professional basketball players. The researchers found that long-term low-dose creatine monohydrate supplementation did not promote clinically significant changes in health markers or side effects. Thus, contrary to unsubstantiated reports, the peer-reviewed literature demonstrates that there is *no evidence* that: 1) creatine supplementation increases the anecdotally reported incidence of musculoskeletal injuries, dehydration, muscle cramping, gastrointestinal upset, renal dysfunction, etc.; or that 2) long-term creatine supplementation results in any clinically significant side effects among athletes during training or competition for up to 3 years. If anything, evidence reveals that athletes who take creatine during training and competition experience a lower incidence of injuries compared to athletes who do not supplement their diet with creatine.

### Enhanced tolerance to exercise in the heat

Like carbohydrate, creatine monohydrate has osmotic properties that help retain a small amount of water. For example, initial studies reported that creatine loading promoted a short-term fluid retention (e.g., about 0.5 – 1.0 L) that was generally proportional to the acute weight gain observed [22, 46]. For this reason, there was interest in determining if creatine supplementation may help hyper-hydrate an athlete and/or improve exercise tolerance when exercising in the heat [76, 115–126]. For example, Volek and colleagues [76] evaluated the effects of creatine supplementation (0.3 g/kg/day for 7 days) on acute cardiovascular, renal, temperature, and fluid-regulatory hormonal responses to exercise for 35 min in the heat. The researchers reported that creatine supplementation augmented repeated sprint cycle performance in the heat without altering thermoregulatory responses. Kilduff and associates [123] evaluated the effects of creatine supplementation (20 g/day for 7 days) prior to performing exercise to exhaustion at 63% of peak oxygen uptake in the heat (30.3 °C). The researchers reported that creatine supplementation increased intracellular water and reduced thermoregulatory and cardiovascular responses to prolonged exercise (e.g., heart rate, rectal temperature, sweat rate) thereby promoting hyper-hydration and a more efficient thermoregulatory response during prolonged exercise in the heat. Watson and colleagues [117] reported that short-term creatine supplementation (21.6 g/day for 7 days) did not increase the incidence of symptoms or compromise hydration status or thermoregulation in dehydrated (−2%), trained men exercising in the heat. Similar findings were observed by several other groups [118, 119, 127, 128] leading researchers to add creatine to glycerol as a highly effective hyper-hydrating strategy to help athletes better tolerate exercise in the heat [116, 120–122, 125, 126]. These findings provide strong evidence that creatine supplementation (with or without glycerol) may serve as an effective nutritional hyper-hydration strategy for athletes engaged in intense exercise in hot and humid environments thereby *reducing* risk to heat related-illness [5, 129].

### Enhanced rehabilitation from injury

Since creatine supplementation has been reported to promote gains in muscle mass and improved strength, there has been interest in examining the effects of creatine supplementation on muscle atrophy rates as a result of limb immobilization and/or during rehabilitation [130]. For example, Hespel and coworkers [131] examined the effects of creatine supplementation (20 g/day down to 5 g/day) on atrophy rates and rehabilitation outcomes in individuals who had their right leg casted for 2 weeks. During the 10 week rehabilitation phase, participants performed three sessions a week of knee extension rehabilitation. The researchers reported that individuals in the creatine group experienced greater changes in the cross-sectional area of muscle fiber (+10%) and peak strength (+25%) during the rehabilitation period. These changes were associated with greater changes in myogenic regulating factor 4 (MRF4) and myogenic protein expression. In a companion paper to this study, Op’t Eijnde et al. [132] reported that creatine supplementation offset the decline in muscle GLUT4 protein content that occurs during immobilization and increased GLUT4 protein content during subsequent rehabilitation training in healthy subjects. Collectively, these findings suggest that creatine supplementation lessened the amount of muscle atrophy and detrimental effects on muscle associated with immobilization while promoting greater gains in strength during rehabilitation. Similarly, Jacobs and associates [133] examined the effects of creatine supplementation (20 g/d for 7 days) on upper extremity work capacity in individuals with cervical-level spinal cord injury (SCI). Results revealed that peak oxygen uptake and ventilatory anaerobic threshold were increased following creatine supplementation. Conversely, Tyler et al. [134] reported that creatine supplementation (20 g/day for 7 days, and 5 g/day thereafter) did not significantly affect strength or functional capacity in patients recovering from anterior cruciate ligament (ACL) surgery. Moreover, Perret and colleagues [135] reported that creatine supplementation (20 g/day for 6 days) did not enhance 800 m wheelchair performance in trained SCI wheelchair athletes. While not all studies show benefit, there is evidence that creatine supplementation may help lessen muscle atrophy following immobilization and promote recovery during exercise-related rehabilitation in some populations. Thus, creatine supplementation may help athletes and individuals with clinical conditions recover from injuries.

### Brain and spinal cord neuroprotection

The risk of concussions and/or SCI in athletes involved in contact sports has become an international concern among sports organizations and the public. It has been known for a long time that creatine supplementation possesses neuroprotective benefits [29, 38, 40, 136]. For this reason, a number of studies have examined the effects of creatine supplementation on traumatic brain injury (TBI), cerebral ischemia, and SCI. For example, Sullivan et al. [137] examined the effects of 5 days of creatine administration prior to a controlled TBI in rats and mice. The researchers found that creatine monohydrate ameliorated the extent of cortical damage by 36 to 50%. The protection appeared to be related to creatine-induced maintenance of neuronal mitochondrial bioenergetics. Therefore, the researchers concluded that creatine supplementation may be useful as a neuroprotective agent against acute and chronic neurodegenerative processes. In a similar study, Haussmann and associates [138] investigated the effects of rats fed creatine (5 g/100 g dry food) before and after a moderate SCI. The researchers reported that creatine ingestion improved locomotor function tests and reduced the size of scar tissue after the SCI. The authors suggested that pretreatment of patients with creatine may provide neuroprotection in patients undergoing spinal surgery who are at risk to SCI. Similarly, Prass and colleagues [139] reported findings that creatine administration reduced brain infarct size following an ischemic event by 40%.

Adcock et al. [140] reported that neonatal rats fed 3 g/kg of creatine for 3 days observed a significant increase in the ratio of brain PCr to Pi and a 25% reduction in the volume of edemic brain tissue following cerebral hypoxic ischemia. The authors concluded that creatine supplementation appears to improve brain bioenergetics thereby helping minimize the impact of brain ischemia. Similarly, Zhu and colleagues [141] reported that oral creatine administration resulted in a marked reduction in ischemic brain infarction size, neuronal cell death, and provided neuroprotection after cerebral ischemia in mice. The authors suggested that given the safety record of creatine, creatine might be considered as a novel therapeutic agent for inhibition of ischemic brain injury in humans. Allah et al. [142] reported that creatine monohydrate supplementation for 10 weeks reduced the infarction size and improved learning/memory following neonatal hypoxia ischemia encephalopathy in female mice. The authors concluded that creatine supplementation has the potential to improve the neuro-function following neonatal brain damage. Finally, Rabchevsky and associates [143] examined the efficacy of creatine-supplemented diets on hind limb functional recovery and tissue sparing in adult rats. Rats were fed a control diet or 2% creatine-supplemented chow for 4–5 weeks prior to and following SCI. Results revealed that creatine feeding significantly reduced loss of gray matter after SCI. These findings provide strong evidence that creatine supplementation may limit damage from concussions, TBI, and/or SCI [33, 144].

## Potential medical uses of creatine

Given the role of creatine in metabolism, performance, and training adaptations; a number of researchers have been investigating the potential therapeutic benefits of creatine supplementation in various clinical populations. The following highlights some of these applications.

### Creatine synthesis deficiencies

Creatine deficiency syndromes are a group of inborn errors (e.g., AGAT deficiency, GAMT deficiency, and CRTR deficiency) that reduce or eliminate the ability to endogenously synthesize or effect transcellular creatine transport [17]. Individuals with creatine synthesis deficiencies have low levels of creatine and PCr in the muscle and the brain. As a result, they often have clinical manifestations of muscle myopathies, gyrate atrophy, movement disorders, speech delay, autism, mental retardation, epilepsy, and/or developmental problems [13, 17, 145]. For this reason, a number of studies have investigated the use of relatively high doses of creatine monohydrate supplementation (e.g., 0.3 – 0.8 g/kg/day equivalent to 21 – 56 g/day of creatine for a 70 kg person, or 1 – 2.7 times greater than the adult loading dose) throughout the lifespan as a means of treating children and adults with creatine synthesis deficiencies [13, 17, 145–149]. These studies generally show some improvement in clinical outcomes particularly for AGAT and GAMT with less consistent effects on CRTR deficiencies [145].

For example, Battini et al. [150] reported that a patient diagnosed at birth with AGAT deficiency who was treated with creatine supplementation beginning at 4 months of age experienced normal psychomotor development at 18 months compared to siblings who did not have the deficiency. Stockler-Ipsiroglu and coworkers [151] evaluated the effects of creatine monohydrate supplementation (0.3 – 0.8 g/kg/day) in 48 children with GMAT deficiency with clinical manifestations of global developmental delay/intellectual disability (DD/ID) with speech/language delay and behavioral problems (*n* = 44), epilepsy (*n* = 35), or movement disorder (*n* = 13). The median age at treatment was 25.5 months, 39 months, and 11 years in patients with mild, moderate, and severe DD/ID, respectively. The researchers found that creatine supplementation increased brain creatine levels and improved or stabilized clinical symptoms. Moreover, four patients treated younger than 9 months had normal or almost normal developmental outcomes. Long-term creatine supplementation has also been used to treat patients with creatine deficiency-related gyrate atrophy [152–156]. These findings and others provide promise that high-dose creatine monohydrate supplementation may be an effective adjunctive therapy for children and adults with creatine synthesis deficiencies [18, 145, 157–159]. Additionally, these reports provide strong evidence regarding the long-term safety and tolerability of high-dose creatine supplementation in pediatric populations with creatine synthesis deficiencies, including infants less than 1 year of age [157].

### Neurodegenerative diseases

A number of studies have investigated the short and long-term therapeutic benefit of creatine supplementation in children and adults with various neuromuscular diseases like muscular dystrophies [160–165], Huntington’s disease [23, 166–171]; Parkinson disease [23, 40, 166, 172–174]; mitochondria-related diseases [29, 175–177]; and, amyotrophic lateral sclerosis or Lou Gehrig’s Disease [166, 178–184]. These studies have provided some evidence that creatine supplementation may improve exercise capacity and/or clinical outcomes in these patient populations. However, Bender and colleagues [23] recently reported results of several large clinical trials evaluating the effects of creatine supplementation in patients with Parkinson’s disease (PD), Huntington’s disease (HD), and amyotrophic lateral sclerosis (ALS). A total of 1,687 patients took an average of 9.5 g/day of creatine for a total of 5,480 patient years. Results revealed no clinical benefit on patient outcomes in patients with PD or ALS. However, there was some evidence that creatine supplementation slowed down progression of brain atrophy in patients with HD (although clinical markers were unaffected). Whether creatine supplementation may have a role in mediating other clinical markers in these patient populations and/or whether individual patients may respond more positively to creatine supplementation than others, remain to be determined. Nevertheless, these studies show that creatine supplementation has been used to treat children and adults with neurodegenerative conditions and is apparently safe and well-tolerated when taking up to 30 g/day for 5 years in these populations.

### Ischemic heart disease

Creatine and phosphocreatine play an important role in maintaining myocardial bioenergetics during ischemic events [33]. For this reason, there has been interest in assessing the role of creatine or phosphocreatine in reducing arrhythmias and/or improving heart function during ischemia [185–194]. In a recent review, Balestrino and colleagues [33] concluded that phosphocreatine administration, primarily as an addition to cardioplegic solutions, has been used to treat myocardial ischemia and prevent ischemia-induced arrhythmia and improve cardiac function with some success. They suggested that creatine supplementation may protect the heart during an ischemic event. Thus, prophylactic creatine supplementation may be beneficial for patients at risk for myocardial ischemia and/or stroke.

### Aging

A growing collection of evidence supports that creatine supplementation may improve health status as individuals age [41, 43–45, 195]. In this regard, creatine supplementation has been reported to help lower cholesterol and triglyceride levels [67, 196]; reduce fat accumulation in the liver [197]; reduce homocysteine levels [198]; serve as an antioxidant [199–202]; enhance glycemic control [132, 203–205]; slow tumor growth in some types of cancers [32, 198, 206, 207]; increase strength and/or muscle mass [37, 41, 44, 45, 82, 208–212]; minimize bone loss [211, 212]; improve functional capacity in patients with knee osteoarthritis [213] and fibromyalgia [214]; positively influence cognitive function [43, 83, 195]; and in some instances, serve as an anti-depressant [215–217].

For example, Gualano and associates supplemented patients with type II diabetes with a placebo or creatine (5 g/day) for 12 weeks during training. Creatine supplementation significantly decreased HbA1c and glycemic response to standardized meal as well as increased GLUT-4 translocation. These findings suggest that creatine supplementation combined with an exercise program improves glycemic control and glucose disposal in type 2 diabetic patients. Candow and others [211] reported that low-dose creatine (0.1 g/kg/day) combined with protein supplementation (0.3 g/kg/day) increased lean tissue mass and upper body strength while decreasing markers of muscle protein degradation and bone resorption in older men (59–77 years). Similarly, Chilibeck et al. [212] reported that 12 months of creatine supplementation (0.1 g/kg/day) during resistance training increased strength and preserved femoral neck bone mineral density and increased femoral shaft subperiosteal width in postmenopausal women. A recent meta-analysis [80] of 357 elderly individuals (64 years) participating in an average of 12.6 weeks of resistance training found that participants supplementing their diet with creatine experienced greater gains in muscle mass, strength, and functional capacity. These findings were corroborated in a meta-analysis of 405 elderly participants (64 years) who experienced greater gains in muscle mass and upper body strength with creatine supplementation during resistance-training compared to training alone [37]. These findings suggest that creatine supplementation can help prevent sarcopenia and bone loss in older individuals.

Finally, a number of studies have shown that creatine supplementation can increase brain creatine content generally by 5 – 15% [218–220]. Moreover, creatine supplementation can reduce mental fatigue [221] and/or improve cognitive function [83, 222–225]. For example, Watanabe et al. [221] reported that creatine supplementation (8 g/day for 5 days) reduced mental fatigue when subjects repeatedly performed a simple mathematical calculation as well as increased oxygen utilization in the brain. Rae and colleagues [222] reported that creatine supplementation (5 g/day for 6 weeks) significantly improved working memory and intelligence tests requiring speed of processing. McMorris and coworkers [224] found that creatine supplementation (20 g/day for 7 days) after sleep deprivation demonstrated significantly less decrement in performance in random movement generation, choice reaction time, balance and mood state suggesting that creatine improves cognitive function in response to sleep deprivation. This research group also examined the effects of creatine supplementation (20 g/day for 7 days) on cognitive function in elderly participants and found that creatine supplementation significantly improved performance on random number generation, forward spatial recall, and long-term memory tasks. Ling and associates [225] reported that creatine supplementation (5 g/day for 15 days) improved cognition on some tasks. Since creatine uptake by the brain is slow and limited, current research is investigating whether dietary supplementation of creatine precursors like GAA may promote greater increases in brain creatine [226, 227]. One recent study suggested that GAA supplementation (3 g/day) increased brain creatine content to a greater degree than creatine monohydrate [227].

### Pregnancy

Since creatine supplementation has been shown to improve brain and heart bioenergetics during ischemic conditions and possess neuroprotective properties, there has been recent interest in use of creatine during pregnancy to promote neural development and reduce complications resulting from birth asphyxia [228–237]. The rationale for creatine supplementation during pregnancy is that the fetus relies upon placental transfer of maternal creatine until late in pregnancy and significant changes in creatine synthesis and excretion occur as pregnancy progresses [230, 232]. Consequently, there is an increased demand for and utilization of creatine during pregnancy. Maternal creatine supplementation has been reported to improve neonatal survival and organ function following birth asphyxia in animals [228, 229, 231, 233–235, 237]. Human studies show changes in the maternal urine and plasma creatine levels across pregnancy and association to maternal diet [230, 232]. Consequently, it has been postulated that there may be benefit to creatine supplementation during pregnancy on fetal growth, development, and health [230, 232]. This area of research may have broad implications for fetal and child development and health.

## Safety

Since creatine monohydrate became a popular dietary supplement in the early 1990s, over 1,000 studies have been conducted and billions of servings of creatine have been ingested. The only consistently reported side effect from creatine supplementation that has been described in the literature has been weight gain [5, 22, 46, 78, 91, 92, 112]. Available short and long-term studies in healthy and diseased populations, from infants to the elderly, at dosages ranging from 0.3 to 0.8 g/kg/day for up to 5 years have consistently shown that creatine supplementation poses no adverse health risks and may provide a number of health and performance benefits. Additionally, assessments of adverse event reports related to dietary supplementation, including in pediatric populations, have revealed that creatine was rarely mentioned and was not associated with any significant number or any consistent pattern of adverse events [238–240]. Unsubstantiated anecdotal claims described in the popular media as well as rare case reports described in the literature without rigorous, systematic causality assessments have been refuted in numerous well-controlled clinical studies showing that creatine supplementation does not increase the incidence of musculoskeletal injuries [22, 111, 112, 241], dehydration [111, 112, 117, 122, 127–129, 242], muscle cramping [76, 106, 111, 112, 117], or gastrointestinal upset [22, 111, 112, 241]. Nor has the literature provided any support that creatine promotes renal dysfunction [22, 51, 85, 114, 156, 172, 243–248] or has long-term detrimental effects [22, 23, 53, 155, 172]. Rather, as noted above, creatine monohydrate supplementation has been found to reduce the incidence of many of these anecdotally reported side effects.

With regard to the question of whether creatine has effect on renal function, a few case studies [249–252] reported that individuals purportedly taking creatine with or without other supplements presented with high creatinine levels and/or renal dysfunction [249–251]. Additionally, one study suggested that feeding rats with renal cystic disease 2 g/kg/d of creatine for 1 week (equivalent to 140 g/day for a 70 kg individual) and 0.4 g/kg/d (equivalent to 28 g/day for a 70 kg individual) for 4 weeks exacerbated disease progression. These reports prompted some concern that creatine supplementation may impair renal function [253–256] and prompted a number of researchers to examine the impact of creatine supplementation on renal function [22, 51, 85, 114, 156, 172, 243–248, 257–259]. For example, Ferreira and associates [260] reported that creatine feeding (2 g/kg/d for 10 weeks equivalent to 140 g/kg/d in a 70 kg person) had no effects on glomerular filtration rate and renal plasma flow in Wistar rats. Likewise, Baracho and colleagues [261] reported that Wistar rats fed 0, 0.5, 1, or 2 g/kg/d of creatine did not result in renal and/or hepatic toxicity. Poortmans and coworkers reported that ingesting 20 g/day of creatine for 5 days [243], and up to 10 g/day from 10 months to 5 years [257] had no effect on creatine clearance, glomerular filtration rate, tubular resorption, or glomerular membrane permeability compared to controls. Kreider et al. [22] reported that creatine supplementation (5–10 g/day for 21 months) had no significant effects on creatinine or creatinine clearance in American football players. Gualono and associates [262] reported that 12 weeks of creatine supplementation had no effects on kidney function in type 2 diabetic patients. Finally, creatine supplement has been used as a means of reducing homocysteine levels and/or improving patient outcomes in patients with renal disease [263–265] as well as ameliorating birth asphyxia related renal dysfunction in mice [228]. Moreover, long-term, high dose ingestion of creatine (up to 30 g/d for up to 5 years) in patient populations has not been associated with an increased incidence of renal dysfunction [23, 155, 156, 172]. While some have suggested that individuals with pre-existing renal disease consult with their physician prior to creatine supplementation in an abundance of caution, these studies and others have led researchers to conclude that there is no compelling evidence that creatine supplementation negatively affects renal function in healthy or clinical populations [5, 6, 22, 53, 259, 266, 267].

Performance-related studies in adolescents, younger individuals, and older populations have consistently reported ergogenic benefits with no clinically significant side effects [5, 6, 22, 23, 53, 113, 129, 244, 245, 268]. The breadth and repetition of these findings provide compelling evidence that creatine monohydrate is well-tolerated and is safe to consume in healthy untrained and trained individuals regardless of age. Moreover, as noted above, the number of potential medical uses of creatine supplementation that can improve health and well-being as one ages and/or may provide therapeutic benefit in clinical populations ranging from infants to senior adults has continued to grow without identifying significant risks or adverse events even in these diseased or compromised special populations. It is no wonder that Wallimann and colleagues [27] recommended that individuals should consume 3 g/day of creatine throughout the lifespan to promote general health.

Some critics of creatine supplementation have pointed to warnings listed on some product labels that individuals younger than 18 years of age should not take creatine as evidence that creatine supplementation is unsafe in younger populations. It’s important to understand that this is a legal precaution and that there is no scientific evidence that children and/or adolescents should not take creatine. As noted above, a number of short- and long-term studies using relatively high doses of creatine have been conducted in infants, toddlers and adolescents with some health and/or ergogenic benefit observed. These studies provide no evidence that use of creatine at recommended doses pose a health risk to individuals less than 18 years of age. Creatine supplementation may, however, improve training adaptations and/or reduce risk to injury, including in younger athletes. For this reason, it is our view that creatine supplementation is an acceptable nutritional strategy for younger athletes who: a.) are involved in serious/competitive supervised training; b.) are consuming a well-balanced and performance enhancing diet; c.) are knowledgeable about appropriate use of creatine; and d.) do not exceed recommended dosages.

## Position of the internationals society of sports nutrition (ISSN)

After reviewing the scientific and medical literature in this area, the International Society of Sports Nutrition concludes the following in terms of creatine supplementation as the official Position of the Society:

1. Creatine monohydrate is the most effective ergogenic nutritional supplement currently available to athletes with the intent of increasing high-intensity exercise capacity and lean body mass during training.
2. Creatine monohydrate supplementation is not only safe, but has been reported to have a number of therapeutic benefits in healthy and diseased populations ranging from infants to the elderly. There is no compelling scientific evidence that the short- or long-term use of creatine monohydrate (up to 30 g/day for 5 years) has any detrimental effects on otherwise healthy individuals or among clinical populations who may benefit from creatine supplementation.
3. If proper precautions and supervision are provided, creatine monohydrate supplementation in children and adolescent athletes is acceptable and may provide a nutritional alternative with a favorable safety profile to potentially dangerous anabolic androgenic drugs. However, we recommend that creatine supplementation only be considered for use by younger athletes who: a.) are involved in serious/competitive supervised training; b.) are consuming a well-balanced and performance enhancing diet; c.) are knowledgeable about appropriate use of creatine; and d.) do not exceed recommended dosages.
4. Label advisories on creatine products that caution against usage by those under 18 years old, while perhaps intended to insulate their manufacturers from legal liability, are likely unnecessary given the science supporting creatine’s safety, including in children and adolescents.
5. At present, creatine monohydrate is the most extensively studied and clinically effective form of creatine for use in nutritional supplements in terms of muscle uptake and ability to increase high-intensity exercise capacity.
6. The addition of carbohydrate or carbohydrate and protein to a creatine supplement appears to increase muscular uptake of creatine, although the effect on performance measures may not be greater than using creatine monohydrate alone.
7. The quickest method of increasing muscle creatine stores may be to consume ~0.3 g/kg/day of creatine monohydrate for 5–7-days followed by 3–5 g/day thereafter to maintain elevated stores. Initially, ingesting smaller amounts of creatine monohydrate (e.g., 3–5 g/day) will increase muscle creatine stores over a 3–4 week period, however, the initial performance effects of this method of supplementation are less supported.
8. Clinical populations have been supplemented with high levels of creatine monohydrate (0.3 – 0.8 g/kg/day equivalent to 21–56 g/day for a 70 kg individual) for years with no clinically significant or serious adverse events.
9. Further research is warranted to examine the potential medical benefits of creatine monohydrate and precursors like guanidinoacetic acid on sport, health and medicine.

## Conclusion

Creatine monohydrate remains one of the few nutritional supplements for which research has consistently shown has ergogenic benefits. Additionally, a number of potential health benefits have been reported from creatine supplementation. Comments and public policy related to creatine supplementation should be based on careful assessment of the scientific evidence from well-controlled clinical trials; not unsubstantiated anecdotal reports, misinformation published on the Internet, and/or poorly designed surveys that only perpetuate myths about creatine supplementation. Given all the known benefits and favorable safety profile of creatine supplementation reported in the scientific and medical literature, it is the view of ISSN that government legislatures and sport organizations who restrict and/or discourage use of creatine may be placing athletes at greater risk—particularly in contact sports that have risk of head trauma and/or neurological injury thereby opening themselves up to legal liability. This includes children and adolescent athletes engaged in sport events that place them at risk for head and/or spinal cord injury.
